# *In vitro* and *in vivo* anti-tumor activity of CoQ_0_ against melanoma cells: inhibition of metastasis and induction of cell-cycle arrest and apoptosis through modulation of Wnt/β-catenin signaling pathways

**DOI:** 10.18632/oncotarget.7983

**Published:** 2016-03-08

**Authors:** You-Cheng Hseu, Varadharajan Thiyagarajan, Hsiao-Tung Tsou, Kai-Yuan Lin, Hui-Jye Chen, Chung-Ming Lin, Jiuun-Wang Liao, Hsin-Ling Yang

**Affiliations:** ^1^ Department of Cosmeceutics, College of Biopharmaceutical and Food Sciences, China Medical University, Taichung 402, Taiwan; ^2^ Department of Health and Nutrition Biotechnology, Asia University, Taichung 41354, Taiwan; ^3^ Institute of Nutrition, China Medical University, Taichung 402, Taiwan; ^4^ Department of Medical Research, Chi-Mei Medical Center, Tainan 710, Taiwan; ^5^ Department of Biotechnology, Ming Chuan University, Taoyuan 333, Taiwan; ^6^ Graduate Institute of Basic Medical Science, China Medical University, Taichung 402, Taiwan; ^7^ Graduate Institute of Veterinary Pathology, National Chung-Hsing University, Taichung 402, Taiwan

**Keywords:** CoQ_0_, melanoma, Wnt/β-catenin, apoptosis, metastasis

## Abstract

Coenzyme Q_0_ (CoQ_0_, 2,3-dimethoxy-5-methyl-1,4-benzoquinone), a novel quinone derivative, has been shown to modulate cellular redox balance. However, effect of this compound on melanoma remains unclear. This study examined the *in vitro* or *in vivo* anti-tumor, apoptosis, and anti-metastasis activities of CoQ_0_ (0-20 μM) through inhibition of Wnt/β-catenin signaling pathway. CoQ_0_ exhibits a significant cytotoxic effect on melanoma cell lines (B16F10, B16F1, and A2058), while causing little toxicity toward normal (HaCaT) cells. The suppression of β-catenin was seen with CoQ_0_ administration accompanied by a decrease in the expression of Wnt/β-catenin transcriptional target c-myc, cyclin D1, and survivin through GSK3β-independent pathway. We found that CoQ_0_ treatment caused G_1_ cell-cycle arrest by reducing the levels of cyclin E and CDK4. Furthermore, CoQ_0_ treatment induced apoptosis through caspase-9/-3 activation, PARP degradation, Bcl-2/Bax dysregulation, and p53 expression. Notably, non- or sub-cytotoxic concentrations of CoQ_0_ markedly inhibited migration and invasion, accompanied by the down-regulation of MMP-2 and -9, and up-regulation of TIMP-1 and -2 expressions in highly metastatic B16F10 cells. Furthermore, the *in vivo* study results revealed that CoQ_0_ treatment inhibited the tumor growth in B16F10 xenografted nude mice. Histological analysis and western blotting confirmed that CoQ_0_ significantly decreased the xenografted tumor progression as demonstrated by induction of apoptosis, suppression of β-catenin, and inhibition of cell cycle-, apoptotic-, and metastatic-regulatory proteins. The data suggest that CoQ_0_ unveils a novel mechanism by down-regulating Wnt/β-catenin pathways and could be used as a potential lead compound for melanoma chemotherapy.

## INTRODUCTION

Melanoma arises from the malignant transformation of pigmented cells of the skin called melanocytes. UV radiation is well documented risk factor for melanoma. Some other risk factor includes atypical nevi, genetic disorders (xeroderma pigmentosum), and fair skin phototype [1]. Malignant melanoma is one of the most deadly forms of skin cancer with increased metastatic potential and high resistance to cytotoxic agents. Melanoma causes 80% of deaths when compared to non-melanoma skin cancers [2]. Although significant progress has been made in melanoma detection and treatment, prognosis for melanoma remains poor [3]. Therefore developing a novel strategic treatments and new antitumor agents for this disease is highly needed.

Wnt/β-catenin signaling pathway plays a pivotal role in both normal cellular response and tumorigenesis [4]. Studies suggest that involvement of the Wnt/β-catenin signaling pathway in the pathogenesis of malignant melanoma [5, 6]. Aberrant activation of the Wnt/β-catenin pathway has been observed in approximately one-third of melanoma, and this subset has a very poor prognosis, understanding Wnt/β-catenin signaling in respect to cancer development and their possible modulators, which could be a promising target for chemoprevention and chemotherapy [7]. The Wnt extracellular signaling pathway controls multiple aspects of development including embryonic axis formation, proliferation, fate specification, tissue architecture, and cell migration [8]. Wnt signaling has been broadly classified as a canonical and non canonical pathway. Canonical pathway is initiated by binding of appropriate Wnt ligands to the Frizzled and LRP-5/6 co-receptors. Wnt binds to Frizzled receptor and inactivates the β-catenin destructive complex comprising APC, Axin, and GSK3β, via the activation of the dishevelled (Dvl) protein. In this event, β-catenin is not targeted for degradation; instead it disassociates from the complex, translocates into the nucleus, and binds to T-cell factor family Tcf/Lef transcription factors to form a heterodimeric complex that activates the transcription of Wnt target genes c-Myc, survivin, cyclin D1, and metalloproteinase (MMP) [9, 10]. Non-canonical pathways, which are Wnt signaling pathways that act as β-catenin independent manner, require Ror2/Ryk coreceptors instead of Lrp5/6. In Wnt/Frizzled interaction promotes the recruitment of Dvl/Dsh, which inturn binds on small GTPase protein called Rac, further this leads to activation of the MAP kinase cascade and subsequently to the activation of AP1-mediated target gene expression [11].

Ubiquinone analogs have been shown to exhibit strong pro- or anti-oxidant properties [12]. It generates functional and structural damages through hyper generation of ROS production [13]. On the contrary, it also shown to prevent oxidative stress-induced cell death [14] by a mechanism that may involve their antioxidant properties. As compared to the other classes of PTP regulators, ubiquinone analogs display noticeable tissue specificity. Studies have confirmed that the regulation of PTP opening and ROS production by ubiquinone analogs changes depending on the studied cell line, (which precludes any extrapolation from results obtained with liver mitochondria). Ubiquinone analogs represent a recently recognized family of PTP regulators [12, 15, 16]. Coenzyme Q_0_ (Coenzyme Q_0_ or Ubiquinone 0) is a redox-active ubiquinone compound that accumulates predominantly in mitochondria. CoQ_0_ was a potent anti-oxidant and inhibits calcium-dependent opening of mitochondrial permeability transition pore (PTP) and CoQ_0_ was a potent inhibitor than all other quinone analogs [17]. Some studies reported the biological activities of CoQ_0_ *in vitro* or *in vivo*. These studies indicate effects of CoQ_0_ on anti-cancer activity against human breast cancer cells through induction of apoptosis and cell-cycle arrest [18]. CoQ_0_ treatment also shown to decrease the cell viability in A549, HepG2, and SW480 cancer cell lines [19], stimulation of insulin secretion in pancreatic islets [20], anti-angiogenic properties [21] and inhibition of oxidative damage in mice blood and tissues [22]. Therefore, the antitumor efficacy of CoQ_0_ was investigated, and the potential mechanisms of CoQ_0_ against melanoma were also examined in both *in vitro* and *in vivo* models in the present study.

## RESULTS

### CoQ_0_ inhibits the viability and colony formation of melanoma cells

**Figure 1:**
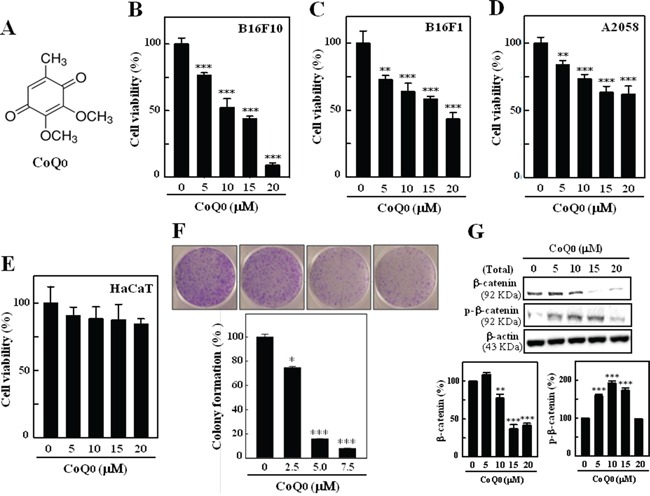
Inhibitory effects on melanoma cell viability and colony formation by CoQ_0_ **A.** Structure of CoQ_0_ (coenzyme Q_0_, 2,3-dimethoxy-5-methyl-1,4-benzoquinone). **B-E.** Murine melanomaB16F10/B16F1, human melanoma A2058, and human keratinocyte HaCaT cells were treated with CoQ_0_ (0-20 μM) or control vehicle for 24 h. Cell viability was determined by MTT assay. **F.** CoQ_0_ inhibits anchorage-independent growth of B16F10 cells. Cells were treated with CoQ_0_ (0-7.5 μM) and assayed for their ability to proliferate and form colonies in soft agar for 5 days. Plating, colonogenic cell survival, and scoring are described in the Materials and Methods. **G.** CoQ_0_ down-regulates β-catenin in B16F10 cells. The total protein levels of β-catenin and p-β-catenin in whole cells were determined by Western blotting. The results are presented as the mean ± S.D of three independent assays. Significant at *p < 0.05; **p < 0.01; ***p < 0.001 compared to untreated control cells.

The effects of (Figure 1A) on the proliferation of murine melanoma cell lines (B16F10, B16F1, and A2058) were investigated. Cells were treated with different concentrations of CoQ_0_ (0-20 μM) for 24 h. To varying extents, a dose-dependent increase in the rate of growth inhibition was observed with 0-20 μM of CoQ_0_. CoQ_0_ treatment for 24 h resulted in a significant (*p*<0.05) cytotoxic effect on B16F10, B16F1, and A2058 melanoma cell lines (Figure 1B-1D). CoQ_0_ treatment showed lesser toxicity on HaCaT cells for 24 h (Figure 1E). These shows CoQ_0_ were more potent to cancer cells than normal cells. Since CoQ_0_ showed a better cytotoxic effect on B16F10 cell line, these cells were used for subsequent experiments. The colony formation ability (a characteristic of tumor cells that is closely correlated with tumorigenesis *in vivo*) was assessed to determine the long-term impact of CoQ_0_ on melanoma cell growth. The colony-forming ability of B16F10 cells was significantly as well as dose-dependently suppressed by CoQ_0_ relative to the controls (Figure 1F). The reductions in colony number were accompanied by a reduction in colony size in B16F10 melanoma cells. These data indicate that treatment of melanoma cells with CoQ_0_ may decrease their rate of proliferation and tumor forming ability.

### CoQ_0_ down-regulates the Wnt/β-catenin signaling pathway in melanoma cells

Dysregulated Wnt/β-catenin signaling pathway and subsequent up-regulation of β-catenin-driven downstream targets c-myc, survivin, and cyclin D1, and MMPs has been detected in a wide range of tumor types, including melanoma [24]. Therefore, we investigated the mechanism of action of growth inhibition by CoQ_0_ in B16F10 melanoma cells. The involvement of Wnt/β-catenin was examined by Western blot. As shown in Figure 1G, CoQ_0_ treatment caused a dose-dependent reduction in the total protein content of β-catenin. However, CoQ_0_ treatment significantly increased β-catenin phosphorylation at serine 33/34 residues, which eventually lead to proteasomal degradation.

### CoQ_0_ suppressed transcriptional activation and nuclear translocation of β-catenin in melanoma cells

**Figure 2:**
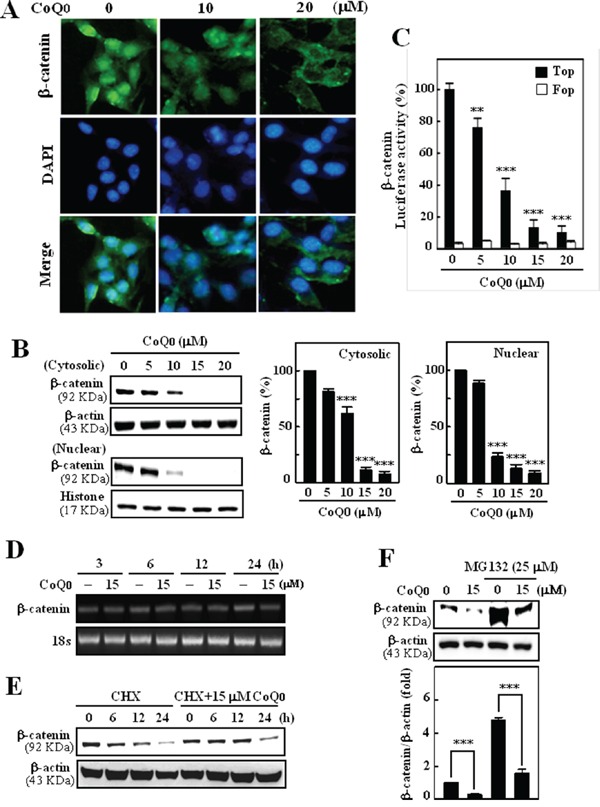
CoQ_0_ suppresses Wnt/β-catenin signaling pathways in melanoma B16F10 cells **A.** Immunocytochemistry was performed to measure the β-catenin expression in B16F10 cells. Cells were grown on 8-well Lab-Tek chambers and treated with CoQ_0_ (10 or 20 μM) for 24 h. Cells were fixed with 2% paraformaldehyde and incubated with specific β-catenin antibodies, followed by a FITC-conjugated secondary antibody (green), and visualized under a confocal microscope. **B.** CoQ_0_ inhibited β-catenin nuclear translocation and transcriptional activation in melanoma cells. Cells were treated with CoQ_0_ (0-20 μM) for 24 h. The levels of β-catenin in the nuclear and cytoplasmic fraction were determined by Western blot. Histone H3 and β-actin were used as an internal loading control, respectively. The photomicrographs shown in this figure are from one representative experiment performed in triplicate, with similar results. **C.** B16F10 cells were transiently transfected with TOP*Flash* or FOP*Flash* plasmids by using lipofectamine, and then incubated with CoQ_0_ (0-20 μM) for 24 h. Cell lysates were mixed with luciferase reagents and quantified by luminometer. Relative β-catenin activity was calculated by dividing the relative luciferase unit (RLU) of treated cells by the RLU of untreated cells. **D.** β-catenin mRNA expression was determined by RT-PCR analyses. Cells were treated with CoQ_0_ (15 μM) for 3, 6, 12, and 24 h. The 18s house keeping gene serves as an internal control. **E.** B16F10 cells were pretreated with Cycloheximide (CHX, 50 μg/mL) for 30 min followed by CoQ_0_ (15 μM) for 6-24 h. **F.** B16F10 cells were pretreated with MG132 (25 μM) for 30 min followed by CoQ_0_ (15 μM) for 24 h. Cell lysates were prepared and assessed by immunoblotting with antibodies to β-catenin. The results are presented as the mean ± SD of three independent assays. Significant at **p < 0.01; ***p < 0.001 compared to untreated control cells.

Transcriptional activation followed by the nuclear translocation of β-catenin is a hallmark of Wnt signaling and is responsible for the transcription of cell growth regulatory genes including c-myc, cyclin D1, and survivin in melanoma cells [25]. Therefore, we performed Western blot and luciferase reporter assays to determine whether the transcriptional activation followed by the nuclear translocation of β-catenin. A similar pattern of results was also observed from the Immunofluorescence assay, indicating that CoQ_0_ treatment dose-dependently inhibited nuclear β-catenin expression in B16F10 melanoma cells (Figure 2A). Furthermore, results of Western blot analyses showed that control cells expressed a greater quantity of β-catenin in both nuclear and cytoplasmic fractions, whereas CoQ_0_ treatment inhibited the accumulation of β-catenin in the nucleus (Figure 2B). The reduction of β-catenin in cytoplasmic fraction was also observed in response to CoQ_0_ treatment (Figure 2B). To further demonstrate that CoQ_0_ modulated the transcriptional activity of β-catenin in melanoma cells, we used the TOP/FOP luciferase reporter system. As shown in Figure 2C, the luciferase activity in B16F10 cells transfected with TOP reporter vector was significantly decreased by CoQ_0_ in a dose-dependent manner, whereas cells transfected with the negative control FOP reporter vector were not affected by CoQ_0_. In contrast, the gene expression pattern of β-catenin mRNA was not affected by CoQ_0_ in B16F10 within the test concentration (Figure 2D). Next, cells were incubated with protein biosynthesis inhibitor (cycloheximide) in the absence or presence of CoQ_0_ (15 μM). The results showed that cells pre-incubated with cycloheximide did not affect the β-catenin level (Figure 2E). Next, we examine whether the degradation of β-catenin by CoQ_0_ is 26S proteasome-dependent, B16F10 cells were incubated with a proteasome-specific inhibitor (MG132) in the absence or presence of CoQ_0_ (15 μM). Western blot analyses showed that cells pre-incubated with MG132 significantly prevented CoQ_0_-induced β-catenin degradation in B16F10 melanoma cells (Figure 2F). Taken together, the above results demonstrate that β-catenin is a bona-fide target of CoQ_0_ in melanoma cells and that CoQ_0_ down-regulated melanoma proliferation by suppression of β-catenin-induced transcriptional activation and nuclear translocation through β-catenin proteasomal degradation.

### CoQ_0_ suppressed β-catenin through GSK3β independent mechanism

**Figure 3:**
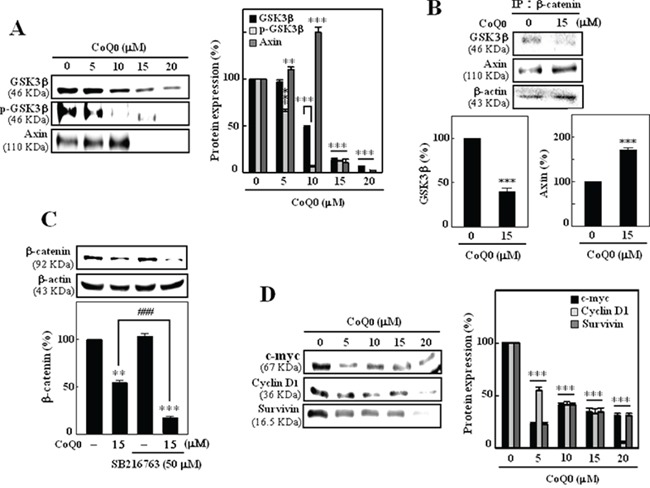
CoQ_0_ inhibits β-catenin through GSK3β-independent pathways in B16F10 cells Cells were pretreated with CoQ_0_ (0-20 μM) for 24 h. **A.** CoQ_0_ down-regulates GSK3β and upregulates Axin in B16F10 cells. The total protein levels of GSK3β, p-GSK3β, and Axin in whole cells were determined by Western blotting. **B.** Equivalent amounts of proteins were immunoprecipitated with anti-GSK3β and anti-Axin antibodies and visualized by Western blot analysis with β-catenin antibodies. **C.** Cells were pre-treated with GSK3β inhibitor SB216763 (50 μM) for 30 min followed by CoQ_0_ (15 μM) for 24 h, β-catenin levels were determined by Western blotting. **D.** Western blotting was performed to measure the expression levels of β-catenin (transcriptional) target genes c-myc, cyclin D1, and survivin in B16F10 cells and then treated with or without CoQ_0_ (0-20 μM) for 24 h. Relative changes in protein bands were measured by densitometric analysis with the control being 100% as shown just below the gel data. Typical results from three independent experiments are shown. The results are presented as the mean ± SD of three independent assays. Significant at **p < 0.01; ***p < 0.001 compared to untreated control cells; significant at ^###^*p* < 0.001 compared to CoQ_0_ alone treated cells.

The up-stream components of β-catenin, including GSK3β, APC, and Axin, form a large multimeric complex that induces phosphorylation and subsequent proteasomal degradation of β-catenin [26]. To further elucidate the role of GSK3β in CoQ_0_-induced down-regulation of β-catenin, the expression levels of GSK3β and Axin were evaluated. Intriguingly, we found that compared to control cells, CoQ_0_ treatment significantly decreased the expression of GSK3β in B16F10 melanoma cells. However, phosphorylation of GSK3β was significantly inhibited by CoQ_0_ treatment, perhaps due to proteasomal degradation. In addition, CoQ_0_ (0-10 μM) treatments dose-dependently increased the expression of axin level and further decreased at higher concentration (Figure 3A). Additionally, lysates were immunoprecipitated with β-catenin and then western blotted for GSK3β and Axin. CoQ_0_ (15 μM) treatment for 24 h decreased the expression of GSK3β and significantly enhanced the expression of Axin level (Figure 3B).

Next, cells were pre-incubated with GSK3β-specific inhibitor SB216763 with or without CoQ_0_ (15 μM). Figure 3C shows that pre-incubation of cells with GSK3β inhibitor did not show any effect on β-catenin expression in B16F10 melanoma cells. However, cells pre incubated with GSK3β inhibitor with CoQ_0_ significantly decreased the expression of β-catenin as compared to CoQ_0_ treatment alone. Thus, these results suggested that GSK3β may not involve in CoQ_0_-induced degradation of β-catenin in B16F10 melanoma cells.

### CoQ_0_ inhibits expression of c-myc, cyclin D, and survivin in melanoma cells

CoQ_0_ significantly inhibited the β-catenin in B16F10 cells; it is logical to speculate that down-regulation of β-catenin's transcriptional targets, including c-myc, cyclin D, and survivin, may be significant evidence of CoQ_0_-induced growth inhibition in melanoma cells. To test this hypothesis, the expression levels of c-myc, cyclin D, and survivin were monitored using Western blot analysis. As shown in Figure 3D, CoQ_0_ down-regulated the expression levels of c-myc, cyclin D, and survivin in a dose-dependent manner in B16F10 cells.

### CoQ_0_ induces apoptosis in melanoma cells

**Figure 4:**
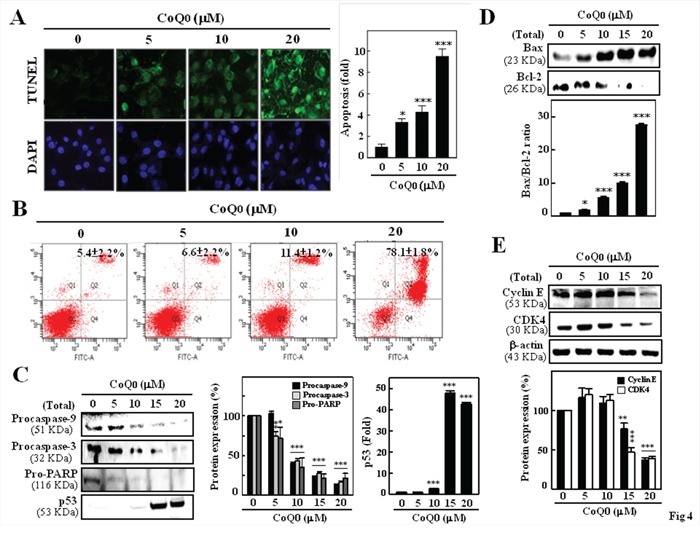
CoQ_0_ induced apoptosis and G1 cell-cycle arrest in melanoma B16F10 cells Cells exposed to CoQ_0_ (0-20 μM) for 24 h. **A.** TUNEL assay was performed to determine CoQ_0_-induced apoptosis by directly measuring DNA fragmentation. A histogram indicates the percentage of apoptotic-positive cells induced by CoQ_0_. **B.** Cells were stained with Annexin V and PI, and analyzed for apoptosis using flow cytometry. Representative flow cytometry patterns are shown. **C-E.** Western blot analysis was performed to measure the expression levels of apoptotic- and cell cycle-related proteins. The effects of CoQ_0_ on the protein levels of Bcl-2, Bax (C), procaspase-3/-9, pro-PARP (D), Cyclin E, CDK4, and p53 (E) in B16F10 cells were monitored with specific antibodies. The results are presented as the mean ± S.D of three independent assays. Significant at *p < 0.05; **p < 0.01; ***p < 0.001 compared to untreated control cells.

The induction of apoptosis (programmed cell death) is a useful approach in cancer theraphy. To assess whether CoQ_0_ promotes apoptosis in melanoma cells, CoQ_0_-induced DNA fragmentation (an apoptotic biomarker) was examined by TUNEL assay. As shown in Figure 4A, CoQ_0_ caused a dose-dependent induction of apoptosis in melanoma cells. At a concentration of 20 μM, apoptotic cells increased by more than 9 fold as compared to control. Next, we examined the effect of CoQ_0_ on B16F10 cell death using Annexin V-FITC/PI staining and flow cytometry (Figure 4B). The Data showed that in control group, 5.4% (late apoptosis) were positive for Annexin V-FITC staining, while CoQ_0_ treatment resulted in 6.6, 11.4, and 78.1% at 5, 10, and 20 μM, respectively. This finding directly correlated with the inhibition of cell growth.

### CoQ_0_-induced apoptosis is mediated by the caspase-dependent mitochondrial pathway

To investigate the signaling cascade which mediates CoQ_0_-induced apoptosis, the pro- and anti-apoptotic proteins were determined by Western blot analysis. To further delineate the activation of caspases, procaspase-9 and -3 were examined. Figure 4C shows that CoQ_0_ treatment caused a significant decrease in the pro-form of caspase-9 and caspase-3 in B16F10 melanoma cells. Treatment of melanoma cells with CoQ_0_ also resulted in a dose-dependent reduction in pro-PARP (Figure 4C). These results collectively suggest that mitochondria- dependent pathway may involve in the CoQ_0_ induced apoptosis of melanoma cells.

Bcl-2 family proteins, including Bcl-2 and Bax, play an important role in the regulation of apoptosis [27]. Thus, we investigated the effect of CoQ_0_ on the expression of anti-apoptotic Bcl-2 and pro-apoptotic Bax in melanoma cells. After 24 h of treatment, CoQ_0_ (20 μM) caused a significant increase in Bax protein level, whereas a dose-dependent reduction in Bcl-2 protein was observed (Figure 4D). The Bax/Bcl-2 ratio in cells can regulate the susceptibility of cells to apoptosis, CoQ_0_ (20 μM) treatment enhanced the Bax/Bcl-2 ratio in B16F10 melanoma cells (Figure 4D). p53, tumor suppressor protein, mediates a variety of anti-proliferative processes through cell cycle checkpoints, and apoptosis [28]. Furthermore, increased p53 (a pro-apoptotic protein) expression was also noted in CoQ_0_-induced B16F10 cells (Figure 4E).

### CoQ_0_ down-regulates cyclin E, and CDK4 in melanoma cells

To further examine the molecular mechanism(s) and underlying changes in cell cycle patterns caused by CoQ_0_ treatment, the expression profile of G_1_/S transition phase regulatory proteins, including cyclin D1/E and their kinase CDK4, was examined by using Western blot. As shown in Figure 4E, CoQ_0_ treatment (0-20 μM) for 24 h caused a dose-dependent reduction of cyclin E and its up-stream kinase, CDK4 in B16F10 melanoma cells. In addition, CoQ_0_ treatment decreased the cyclin D1 expression in a dose dependent manner (Figure 3D). Taken together, these data suggest that CoQ_0_ treatment also promotes cell growth inhibition by inducing G_1_/S transition phase arrest, followed by the down-regulation of cyclin D1/E and CDK4 expression in melanoma cells.

### CoQ_0_ inhibits melanoma migration and invasion *in vitro*

**Figure 5:**
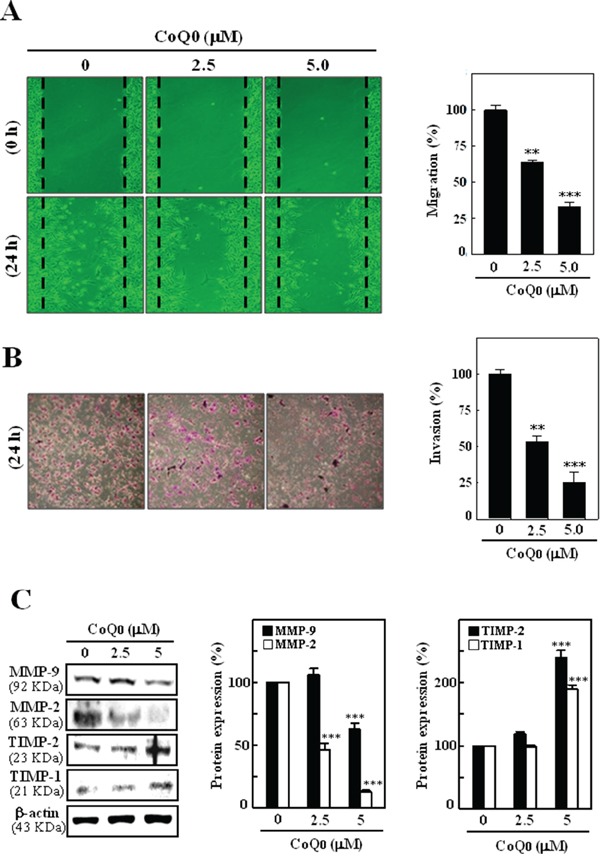
CoQ_0_ inhibits the migration and invasion in melanoma B16F10 cells Cells were treated with CoQ_0_ (0-5 μM) or vehicle control (0.1% DMSO). **A.** Cell migration was observed using a phase-contrast microscope (100× magnification) at 0 and 24 h, and the closure of area was calculated. The percentage of migrated cells was quantified and expressed relative to untreated cells (control), which represented 100%. To quantify migration, cells were counted in three microscopic fields per sample. **B.** After 24 h CoQ_0_ treatment, cells invading under the membrane were photographed (200× magnification). The inhibition of invading cells were quantified and expressed on the basis of untreated cells (control) that represented 100%. **C.** Cells were treated with CoQ_0_ (0-5 μM) for 24 h. Cells mediated the down-regulation of MMP-9 and -2 and up-regulation of TIMP-1 and -2 expressions were monitored by western blot. β-actin was used as a internal control. Relative changes in protein bands were measured using densitometric analysis with the control being 100%. The results are presented as the mean ± S.D of three independent assays. Significant at **p < 0.01; ***p < 0.001 compared to untreated control cells.

To determine the anti-migratory properties of CoQ_0_, the highly metastatic murine melanoma B16F10 cells lines were subjected to an *in vitro* wound healing assay. As shown in Figure 5A, the migration ability of melanoma cells was significantly restricted by CoQ_0_ (0-5 μM). To further examine the possible role of CoQ_0_ in the prevention of melanoma invasion, B16F10 cells were treated with CoQ_0_ (0-5 μM) for 24 h, and the matrigel-based trans-well invasion assay was performed. Treatment of melanoma cells with CoQ_0_ significantly inhibited melanoma invasion (Figure 5B). It must be noted that the melanoma migration and invasion assays were performed with non-cytotoxic or sub-cytotoxic concentrations of CoQ_0_.

### CoQ_0_ down-regulates MMP-2/-9 and up-regulates TIMP-1/-2 expression in melanoma cells

Over expressions of MMPs including MMP-9 and MMP-2 plays a pivotal role in melanoma migration and invasion by stimulating degradation of the extracellular matrix. Therefore, we examined whether the anti-invasive potential of CoQ_0_ (0-5 μM) was associated with down-regulation of MMP-2 and MMP-9 expression. As shown in Figure 5C, CoQ_0_ treatment inhibited the expression of MMP-2 and MMP-9 in a dose-dependent manner. The tissue inhibitors of metalloproteinases (TIMPs) can control MMP activities. Therefore, it was of interest to examine whether CoQ_0_ (0-5 μM) treatment could upregulate TIMPs expression in melanoma cells. Figure 5C shows that as compared to control cells CoQ_0_ treatment enhanced the TIMP-1 and TIMP-2 expressions in B16F10 melanoma cells.

### β-catenin siRNA enhances the anti-tumor effects of CoQ_0_

**Figure 6:**
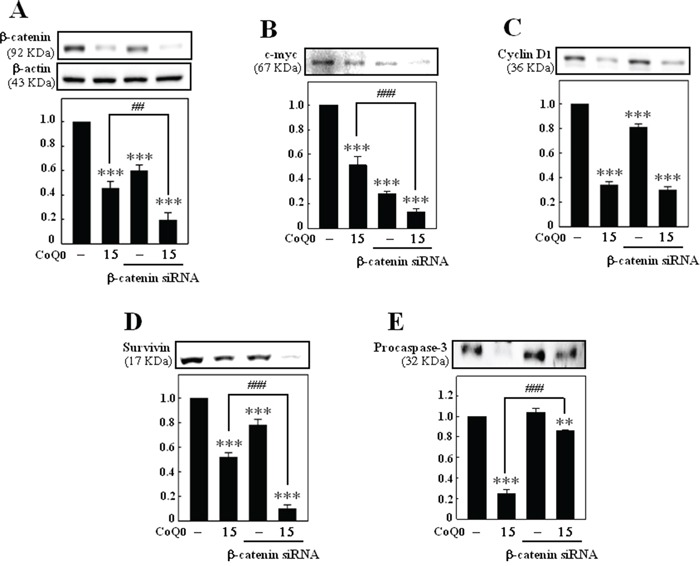
β-catenin siRNA enhances the anti-tumor effects of CoQ_0_ B16F10 cells were transfected with a specific siRNA against β-catenin or a non-silencing control. Following transfection for 24 h, the cells were incubated with or without CoQ_0_ (15 μM for 24 h). The knockdown was evaluated by Western blotting. The expressions of β-catenin **A.** c-myc **B.** cyclin D1 **C.** survivin **D.** and procaspase-3 **E.** were monitored. Relative changes in protein bands were measured by densitometric analysis with the control being 100%. The results are presented as the mean ± S.D of three independent assays. Significant at **p < 0.01; ***p < 0.001 compared to untreated control cells; significant at ^###^*p* < 0.001 compared to CoQ_0_ alone treated cells.

To examine whether CoQ_0_ inhibits c-myc, cyclin D1, survivin, and procaspase-3 through β-catenin signaling, the direct effect of β-catenin siRNA was determined. B16F10 cells were transfected with siRNA and CoQ_0_ for 24 h. Transfection with β-catenin siRNA effectively suppressed the protein expression of β-catenin, c-myc, cyclin D1, and survivin (Figure 6A-6D). However, CoQ_0_ dramatically enhanced the suppression of β-catenin, c-myc, cyclin D1, and survivin, expression in cells transfected with β-catenin siRNA (Figure 6A-6D). Intriguingly, cells transfected with β-catenin siRNA did not show any changes in the expression of procaspase-3. Whereas, cotreatment with CoQ_0_ increased the expression of procaspase-3 level in B16F10 melanoma cells as compared to CoQ_0_ treatment alone (Figure 6E). These results exhibited that CoQ_0_ may have a direct effect on β-catenin signaling pathway.

### *In vivo* inhibition of xenografted growth by CoQ_0_

**Figure 7:**
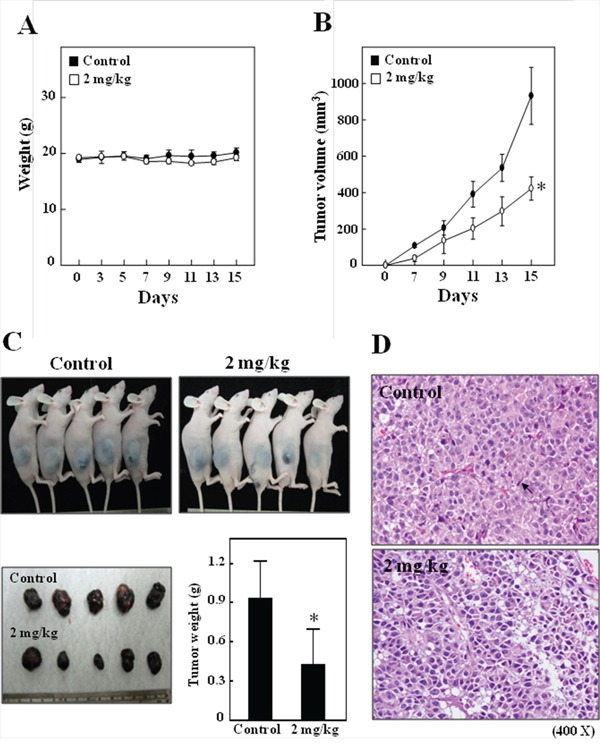
*In vivo* inhibition of B16F10 xenografted proliferation by CoQ_0_ **A-B.** Time-course effect of CoQ_0_ on growth of B16F10 xenografted nude mice was evaluated by measurements of body weight (A), and tumor volume (B) every 2 days. B16F10 cells were implanted subcutaneously into the flanks of nude mice on day 0, and animals were subsequently treated with 2 mg/kg of CoQ_0_ or vehicle (control). **C.** On the 15^th^ day after tumor implantation, animals were photographed. Results are presented as mean ± SE (n=5). **D.** Histochemical analysis of proliferation in B16F10 xenografted tumors. Control and B16F10 xenografted tumors following CoQ_0_ (2 mg/kg) treatments were examined using light microscopy (20× and 200× magnification). Arrows indicate mitotic (tumor control) and pyknotic tumor cells (CoQ_0_). Significant at *p < 0.05 compared to untreated control cells.

Nude mice were used to evaluate the *in vivo* effects of CoQ_0_ on tumor growth. B16F10 cells were xenografted into nude mice. All animals appeared healthy, with no loss of body weight noted during CoQ_0_ treatment (Figure 7A). In addition, no signs of toxicity were observed in any of the nude mice (body weight and microscopic examination of individual organs; data not shown). The time course for B16F10 xenografted tumor growth with CoQ_0_ (2 mg/kg/every 2 days) or with vehicle only (control), is shown in Figure 7B. Evaluation of tumor volume showed a significantly time-dependent growth inhibition associated with CoQ_0_ treatment. Tumor volume in the CoQ_0_-treated mice was inhibited compared with the control group (Figure 7C). At the end of 15 days, the B16F10 xenografted tumor was excised from each sacrificed animal. Additionally, microscopic examination of tumor sections was done to distinguish differences in nucleic and cytoplasmic morphology after 15 days of CoQ_0_ treatment. As shown in Figure 7D, the histopathological findings from inoculated melanoma cells in tumor control nude mice presented newly formed blood vessels with massive necrosis in the area of the tumor mass. Tumor cells were large, round to oval in shape with predominant nucleoli, and expressed high levels of cellular activity and mitotic figures (Figure 7D). In contrast, tumors in the CoQ_0_-treated nude mice showed less angiogenesis, had smaller cells with shrunken, condensed and pyknotic nuclei, indicating tumor cell inactivity or regression (Figure 7D). Interestingly, while abundant mitosis was observed in the proliferating cells in the control group, few mitotic cells were seen in sections from CoQ_0_-treated animals (Figure 7D). Analysis of our data suggests that CoQ_0_ promoted antitumor activity in nude mice bearing B16F10 melanoma xenografts.

### Induction of apoptotic DNA fragmentation by CoQ_0_ in xenografted tumors

**Figure 8:**
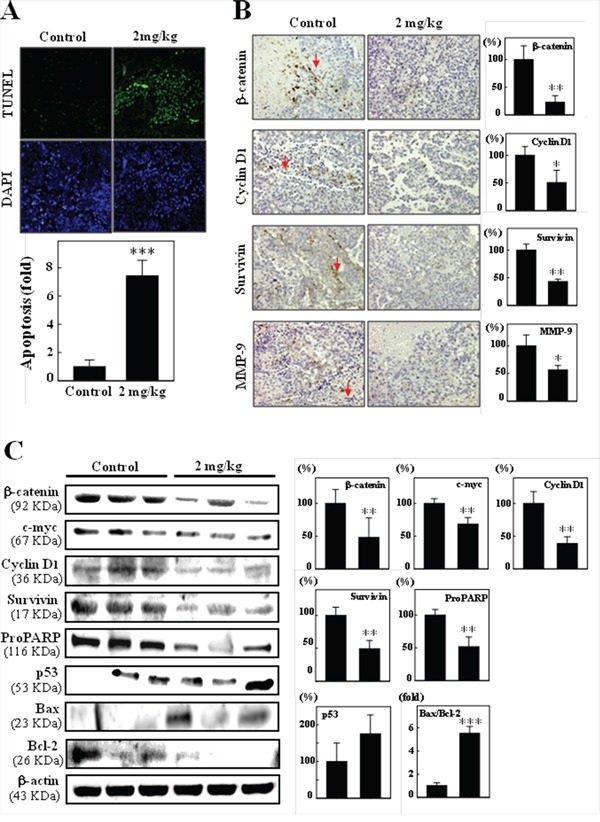
Immunohistochemical staining and western blotting of proliferation, apoptosis, and migration/invasion by CoQ_0_ in B16F10 xenografted tumors **A.** *In situ* apoptosis detection using TUNEL staining in tumor sections from control animals and experimental analogues treated with CoQ_0_ (2 mg/kg). Arrow indicates example apoptotic-positive cells (400 × magnifications). The number of apoptotic-positive cells in microscopic fields from 3 samples was averaged. **B.** Xenografted tumor sections were subjected to immunohistochemical analysis for β-catenin, cyclin D1, survivin, and MMP-9. Cells positive for β-catenin, cyclin D1, survivin, and MMP-9 were counted from 3 fields (200× magnification) for each tumor sample. The number of positive cells (arrows indicate proliferating cells) in microscopic fields from 5∼7 samples was averaged. Results are the mean (±SE) number of cells/microscope field (as percentage) for 5∼7 animals per group. **C.** Western blotting results showing the effects of CoQ_0_ on the total protein contents of β-catenin, c-myc, cyclin D1, survivin, p53, proPARP, Bcl-2, and Bax in the xenografted tumors from 3 samples. Relative changes in protein bands were measured by densitometric analysis with the control being 100% as shown just below the gel data. Significant at *p < 0.05; **p < 0.01; ***p < 0.001 compared to untreated control cells.

The effect of CoQ_0_ on tumor growth (apoptosis) in the B16F10 xenografted mice was also examined using the TUNEL assay on tumor sections. Figure 8A show that there were more TUNEL-positive cells in tumors from CoQ_0_-treated animals, compared to untreated controls (*p* <0.05), which demonstrates that CoQ_0_ treatment was associated with decreased proliferation and increased apoptosis in the study animals. Next, we examined the effect of CoQ_0_ on the targets of β-catenin, cyclin D1, survivin, and MMP-9 by immuhistochemicals analysis of B16F10 melanoma xenografted tumor tissues. Expression of β-catenin, Cyclin D1, Survivin, and MMP-9 was greatly suppressed by treatment with CoQ_0_ (Figure 8B). In addition, western blot analyses also reveal that significant decrease in β-catenin, c-myc, survivin, cyclin D1, and pro-PARP expression, and increase p53 expression and Bax/Bcl-2 ratio in CoQ_0_-treated mice (Figure 8C). These data suggested that CoQ_0_ inhibited B16F10 melanoma tumor development by suppressing Wnt/β-catenin signaling.

## DISCUSSION

*Antrodia camphorata* (AC) is well known in Taiwan as a physiologically beneficial mushroom. There is increasing evidence that AC possesses an extensive range of biological activities, including antioxidant, hepatoprotective, anti-hypertensive, anti-hyperlipidemic, immunomodulatory, and anti-inflammatory properties [29, 30]. AC also exerted potent anti-cancer activity against a variety of cancer cells, including breast, liver, bladder, prostate, oral, colon, lung, pancreatic and leukemic cells [30]. The inhibitory effect against cancer cells by AC may be mediated by various cellular mechanisms of actions, such as regulation of oncogene and tumor suppressor gene expression, inhibition of metastasis and angiogenesis regulatory proteins, down-regulation of signal transduction pathways involving NF-κB, AP-1, Nrf2, and MAPK, induction of cell-cycle arrest, and apoptosis involving the Wnt/β-catenin, p53, death ligands, Bcl-2 and caspase families [23, 29, 30]. CoQ_0_ a major active constituent of AC, significantly inhibited cell growth through the generation of ROS, suppression of HER-2*/neu* signaling, and disruption of the PI3K/Akt-dependent pathway in HER-2*/neu*-overexpressing cells [23]. Despite the emerging evidence of its chemopreventive or chemotherapeutic importance, to date there have been no studies reporting the anti-cancer potential of CoQ_0_against melanoma cells. In the present study, we addressed a novel mechanism of action of CoQ_0_in inducing apoptosis, anti- invasive and anti- migratory effect via modulation of Wnt/β-catenin signaling pathway.

Increasing evidence indicates that Wnt/β-catenin pathway promotes proliferation and cell survival in various normal and cancer cell types, including melanoma cells [31]. Previous studies have demonstrated that down-regulation of the Wnt/β-catenin or Wnt-1 pathway by small interfering RNAs (siRNA) or Wnt-1-targeted monoclonal antibodies induces apoptosis in a variety of human cancer cells. Meanwhile, activation of this pathway is inhibited by chemotherapy-induced apoptosis [10, 32, 33], suggesting that the Wnt/β-catenin pathway may be associated with cellular apoptosis. Melanoma metastasis is often associated with activation of the Wnt/β-catenin signaling pathway [34]. In addition, c-myc was identified as one of the transcriptional targets of β-catenin/Tcf in various cancer cells; suggesting that Wnt signaling functions in oncogenesis, in part, occur through the growth-promoting activity of c-myc [35]. On the other hand, survivin, a member of the inhibitor of apoptosis (IAP) gene family, is an emerging and therapeutic target in most cancer cells [36]. Further, survivin has been correlated with tumor aggression and a poor prognosis for many cancers including melanoma [37]. In the present study we show that CoQ_0_ treatment decreased the β-catenin expression through luciferase assay, immunofluorescence assay, and western blot in B16F10 melanoma cells. This study also elucidated that CoQ_0_ treatment further suppressed the expression of β-catenin downstream target genes, such as c-myc, cyclin D1, and survivin in both *in vitro* and *in vivo*. We suggest that CoQ_0_ exhibit anti-proliferative effect through modulating β-catenin and its downstream target genes, c-myc, cyclin D1, and survivin in B16F10 melanoma cells.

GSK3 act as a tumor suppressor, increased GSK3β protein stability suppressed the Wnt/β-catenin pathway by phosphorylating beta catenin which leads in the ubiquitin/proteosome dependent degradation of β-catenin in melanoma cells [38]. Recently, natural compound have been shown to inhibit the proteasome activity of GSK3β and promote β-catenin degradation [39]. In contrary, aberrant expression of GSK3β has been seen in various types of cancer such as liver, colon, ovarian, and pancreatic cancers [40, 41]. Our present data also provide evidence that GSK3β is not an upstream target of β-catenin expression and is responsible for β-catenin inhibition by CoQ_0_. The effects of CoQ_0_ on GSK3β-independent transcription may be important for CoQ_0_-induced Wnt/β-catenin anti-tumorigenesis. Axin is a multidomain scaffold protein that negatively regulates Wnt signalling pathway [42]. Recently, some biochemical and structural studies have elucidated that Axin binds to β-catenin at a site on armadillo repeats 3-5 and that Phe253 and Lys292 of β-catenin contribute to this interaction [43]. However, the molecular mechanisms by which axin exerts its negative effects has not been elucidated. In the present study, we demonstrate that CoQ_0_ treatment and lysate immunoprecipitated with β-catenin significantly enhanced the axin expression level in B16F10 melanoma cells. These findings suggest that axin may function as a docking station facilitating the interaction of beta-catenin, thereby regulating the Wnt signalling pathway by inducing the down regulation of beta catenin.

Recently several studies pay more attention to cell cycle regulation mediated apoptosis and regard as much effective way to inhibit cancer cell growth [44]. Many apoptotic stimuli induce cell cycle arrest before cell death, thereby affecting both cell cycle and apoptotic machinery. Dysregulation of the cancer cell cycle is one of the therapeutic targets for the development of new anticancer agents [45]. G1 phase is subtly regulated by cyclin/CDK complexes. During G1 phase progression, cyclin D1/CDK4/6 complexes are activated by mid-G1, whereas cyclin E/CDK2 complexes are involved for G1/S transition. Over expression of cyclin D is associated with metastasis and tumorigenesis [46]. In this study, CoQ_0_ led to a sustained suppression of cyclin D1 and cyclin E levels, a result consistent with the inhibition of G_1_/S transition. Moreover, the suppression of cyclin D1 and cyclin E by CoQ_0_ led to the inhibition of CDK4 and CDK2 levels in melanoma cells. Apoptosis is an active mode of cell death, characterized by a number of well-defined features, including cellular morphological changes, chromatin condensation, internucleosomal DNA cleavage and the activation of caspase cascades [47]. The death-receptor-dependent (extrinsic) pathway and mitochondrial-dependent (intrinsic) pathway is two central pathway resulting in cell apoptosis [48]. In the present study, TUNEL assays exhibited that treatment of melanoma cells with CoQ_0_ markedly induced internucleosomal DNA fragmentation, which directly indicates apoptotic cell death. Caspases belongs to family of cysteine acid proteases, play a major role in cell apoptosis. Activation of caspase-9 leads to activation of caspase-3 resulting in a cascade of caspase activity and cell disruption. We demonstrated that CoQ_0_ treatment decreased the procaspase-9 and caspase-3 expression in melanoma cells. Moreover, PARP, a nuclear protein, was shown to be required for apoptosis to proceed in various cell lines. Activated Caspase-3 cleaves PARP (116 kDa), generating 89 kDa inactive fragment and cause apoptosis [49]. In this study, we also found that CoQ_0_ treatment significantly reduced the pro-form of PARP which demonstrated that CoQ_0_ could act as a chemopreventive agent with respect to inhibition of the growth of melanoma cells through the induction of cell cycle arrest and apoptosis.

In mammalian cells, members of the Bcl-2 gene family contains a number of anti-apoptotic proteins, including Bcl-2 and Bcl-xL, which are thought to be involved in resistance to conventional cancer treatment, while the pro-apoptotic proteins from the same gene family, including Bax, Bak and Bad, may induce apoptotic cell death [50]. A hallmark of DNA damage-triggered apoptosis is reduced Bcl-2 expression and increased Bax expression. Therefore, apoptosis largely depends on the balance between anti-apoptotic and pro-apoptotic protein levels. p53 is a tumor suppressor protein plays a substantial role in apoptosis by increasing the transcriptional activity of pro-apoptotic genes such as Bax or decreasing the activity of the anti-apoptotic genes of the Bcl-2 family [51]. Similarly, the present study indicates a dose-dependent inhibition of the anti-apoptotic protein Bcl-2 and a concomitant increase in the expression of the Bax, and p53 proteins by CoQ_0_ in melanoma cells. Therefore, we logically speculated that CoQ_0_ induced its apoptotic effect possibly by up-regulating p53, Bax expression and downregulating Bcl-2 expression.

Metastasis and cell invasion are interrelated processes involving cell migration, growth, adhesion, and proteolytic degradation of tissue barriers such as the extracellular matrix and basement membrane. Among various MMPs, MMP-9 and MMP-2 play crucial roles in tumor cell metastasis and invasion by degradation of type IV collagen, a major component of the ECM [52]. MMPs are naturally found in a complex with their natural inhibitors, known as tissue inhibitors of metalloproteinases (TIMPs), but those are found to be suppressed in metastatic melanoma cell lines [53]. Melanoma cells derived from Wnt, acting through Fz receptors, induce MMP-9 and MMP-2 expression, which plays a vital role in cell migration and invasion [54]. Thus, MMP-2 and MMP-9 can be a target of melanoma cancer therapy by suppressing melanoma cancer invasion. In the present study we show that CoQ_0_ treatment significantly inhibits melanoma migration and invasion by down-regulating MMP-2, MMP-9 and up-regulating TIMP-1 and TIMP-2 expressions. Therefore, CoQ_0_ may inhibit melanoma metastasis through the suppression of MMP-2, and MMP-9 expression.

## MATERIALS AND METHODS

### Reagents and Abs

Dulbecco's modified Eagle's medium (DMEM), fetal bovine serum (FBS),_L_-glutamine and penicillin/streptomycin/neomycin were obtained from GIBCO BRL/Invitrogen (Carlsbad, CA, USA). Anti-rabbit MMP-2, anti-goat MMP-9, anti-mouse Bax, anti-mouse β-actin, anti-rabbit c-myc, anti-rabbit survivin, anti-rabbit Bcl-2, anti-mouse β-catenin, anti-rabbit p53, anti-rabbit caspase-3, antibodies were purchased from Santa Cruz Biotechnology, Inc. (Heidelberg, Germany). Anti-mouse cyclin D1, anti-rabbit PARP, anti-mouse caspase-3/-9, anti-mouse CDK4, anti-rabbit GSK3β, anti-rabbit p-GSK3β, anti-rabbit p-β-catenin and anti-rabbit histone H3 antibodies were obtained from Cell Signaling Technology, Inc. (Danvers, MA, USA). Protease inhibitor MG132 and GSK3β inhibitor SB216763 were purchased from Merk KGaA (Darmstadt, Germany). 3-(4,5-dimethylthiazol-2-yl)-2,5-diphenyltetrazolium bromide (MTT), Cycloheximide, was purchased from Sigma-Aldrich Chemical Co. (St. Louis, MO, USA). CoQ_0_ (Coenzyme Q_0_, 2,3 dimethoxy-5-methyl-1,4 benzoquinone) were purchased from Sigma-Aldrich (St. Louis, MO). All other chemicals were of the highest grade commercially available and were supplied either by Merck or Sigma.

### Cell culture and sample treatment

The murine melanoma (B16F10 and B16F1), human melanoma (A2058), human keratinocyte (HaCaT) cell lines were obtained from the American Type Culture Collection (Manassas, VA, USA). These cells were grown in DMEM supplemented with 10% heat-inactivated FBS, 2 mM L-glutamine, and 1% penicillin-streptomycin-neomycin at 37°C in a humidified incubator with 5% CO_2_.

### MTT assay

The 3-(4,5-dimethylthiazol-2-yl)-2,5-diphenyltetrazolium bromide (MTT, Invitrogen, Grand Island, NY) is a colorimetric based assay that is performed to analyze the proliferation of cells. Briefly, cells (5 × 10^4^ cells/well in 24-well plates) were treated with various concentrations of CoQ_0_ (0-20 μM) for 24 h, after incubation 400 μL 0.5 mg/mL MTT in PBS was added to each well and further incubated for 4 h. The media was removed, and an equal volume of 90% isopropanol and 0.5% SDS mixture (400 μL) was added to dissolve the MTT formazan crystals, and the absorbance was measured at 570 nm (A_570_) using an ELISA microplate reader (μ-Quant, Winooski, VT, USA). The percentage (%) of cell viability was calculated as: (A_570_ of treated cells/A_570_ of untreated cells) × 100.

### Colony formation assay

Anchorage-independent growth was determined by colony formation using the soft agar method. The assay was performed in 6-well plates with a base layer containing 0.5% agar in DMEM containing 10% FBS, 1 mM glutamine, and 100 units of penicillin plus 100 μg/mL of streptomycin. This layer was overlaid with a second layer of 1 mL of 0.35% agar (in DMEM containing 10% FBS, 1 mM glutamine, and 100 units of penicillin plus 100 μg of streptomycin) with a suspension of 1 × 10^4^ cells/well. Fresh medium with CoQ_0_ (0-7.5 μM) was then added to the plates for 24 h. The plates were incubated at 37°C for 5 days, and the tumor colonies were determined with a microscope. The numbers of colonies >200 μm in size were counted using an electron microscope (40 × magnification). Colonies were subsequently stained with p-iodonitrotetrazolium violet (1 mg/mL), and colonies larger than 200 μm were counted. The percentage of colony formation was calculated by defining the number of colonies in the absence of CoQ_0_ as 100%.

### Fluorescent imaging of β-catenin

Cells were seeded at a density of 2×10^4^ cells/well in 8-well Lab-Tek chamber and treated with different concentrations of CoQ_0_ (0-20 μM) for 24 h. After treatment, cells were fixed in 2% paraformaldehyde for 15 min, permeabilized with 0.1% Triton X-100 for 10 min, and then incubated for 1 h with anti-β-catenin primary antibodies in 1.5% FBS. FITC (488 nm) secondary antibodies were incubated for another 1 h in 6% BSA. 1 μg/mL DAPI was stained for 5 min. Stained cells were washed with PBS and visualized using a fluorescence microscope at 400 × magnification.

### Western blot analysis

Cells were seeded in a 6 cm dish at a density of 1 × 10^5^ cells/dish. Next, the cells were treated with or without desired concentrations of CoQ_0_ (0-20 μM) for 24 h. Cells were collected and homogenized in a protein lysis solution (10 mM Tris-HCl [pH 8], 0.32 M sucrose, 1% Triton X-100, 5 mM EDTA, 2 mM dithiothreitol, and 1 mM phenyl methyl sulfonyl fluoride). Proteins were separated by SDS–polyacrylamide gel electrophoresis (SDS-PAGE) and subsequently transferred to PVDF membranes (NENTM Life Science Products, Boston, MA, USA). The blots were blocked with 5% non-fat milk in TBST saline (20 mM Tris–HCl, pH 7.4, 137 mM NaCl, and 0.05% Tween-20) at room temperature (RT) for 1 h and incubated with the appropriate primary antibody at 4°C overnight. The membranes were then incubated with a horseradish peroxidase-conjugated goat anti-rabbit or anti-mouse antibody for 2 h before development using a chemiluminescence substrate (Millipore, Billerica, MA, USA). Densitometry analyses were performed using commercially available quantitative software (AlphaEase, Genetic Technology Inc. Miami, FL) with the control representing 1.0-fold as shown below the data.

### Immunoprecipitation

For immunoprecipitation, 1 mg of protein samples were precleared with protein A-sepharose beads for 1 h and then incubated with 2 mg of anti-β-catenin antibody for 4 h. Immunoprecipitated complex were washed 5 times with RIPA buffer and denatured with SDS sample buffer. The immunoprecipitated product or the total cell lysate (50 mg) were separated by SDS-PAGE, and electrophoretically transferred to PVDC membrane. After blotting with 5% skim milk for 30 min, the membrane was incubated with specific primary antibodies for 2 h, and further incubated with HRP-conjugated secondary antibodies for 1 h. The plots were visualized using ECL reagents (Millipore).

### RT-PCR analysis

Cells were seeded at a density of 4 × 10^6^ cells/dish in 6 cm dish. After reaching 90% confluence, cells were incubated with CoQ_0_ (10 μM) for various time points (0.5-18 h). Total RNA from cultured cells was prepared using the TRIzol reagent (Invitrogen, Grand Island, NY). A 1 μg sample of total RNA was subjected to RT-PCR using a BioRad iCycler PCR instrument (Bio-Rad, Hercules, CA) and the SuperScript-III^®^ One-Step RT-PCR Platinum *taq*^®^ Kit (Invitrogen); amplification was performed in 30-38 cycles at 94°C for 45s (denaturing), 60-65°C for 45s (annealing), and 72°C for 1 min (primer extension). The sequences of the primers used in this study were as follows; β-catenin forward: 5′-TTACCTTCCCGAACATCGAC-3′, reverse: 5′-GCATAAATTCCCACTGCCAC-3′. The PCR products were electrophoresed in a 1% agarose gel and stained with ethidium bromide.

### Luciferase activity assay

To determine the transcriptional activity of β-catenin/TCF, a luciferase reporter assay was performed using the TCF reporter constructs TOP*Flash* and FOP*Flash* as previously. Briefly, cells (5 × 10^4^cells/well) were seeded in 24 well plates and transfected with either TOP*Flash* or FOP*Flash* (100 ng) and the initial control plasmid pRL-TK (5 ng) using lipofectamine™ 2000 reagent (Invitrogen). TOP*Flash* and FOP*Flash* contain wild-type and mutated β-catenin/TCF binding sites, respectively, as well as the thymidine kinase (TK) minimal promoter upstream of the firefly luciferase open reading frame. After transfection, cells were treated with CoQ_0_ (0-20 μM) for 24 h. Cells were then lysed in 350 μL of Triton lysis buffer (50 mM Tris-HCl, 1% (v/v) Triton X-100, 1 mM dithiothreitol, pH 7.8) and centrifuged at 12,000 × *g* for 2 min at 4°C. Luciferase activity was measured by mixing 20 μL of cell lysate with 20 μL of luciferase reagent (470 μM luciferin, 33.3 mM dithiothreitol, 270 μM coenzyme A, 530 μM ATP, 20 mM Tricine, 1.07 mM (MgCO_3_)_4_·Mg(OH)_2_, 2.67 mM MgSO_4_, 0.1 mM EDTA, pH 7.8) and determined with a luminometer (FB15, Zylux Corp., Maryville, TN). Relative β-catenin activity was calculated by dividing the relative luciferase unit (RLU) of treated cells by the RLU of untreated cells.

### Determination of apoptosis

Apoptotic cell death was measured using terminal deoxynucleotidyl transferase-mediated dUTP-fluorescein nick end labeling (TUNEL) with the fragmented DNA detection kit (Roche, Mannheim, Germany) as previously described [23].

### Cell cycle analysis

Cellular DNA content was determined by flow cytometry using the propidium iodide (PI)-labeling method as described previously [23]. Briefly, B16F10 cells were seeded at a density of 4 × 10^5^ cells/dish in 10 cm dishes, and the cell cycle was synchronized by the addition of double thymidine (3 mM) for 16 h. Cell cycle-synchronized cells were then washed with PBS and re-stimulated to enter the G1 phase together by the addition of fresh medium, which also contained various concentrations of CoQ_0_ (0-20 μM). Cells were harvested at 24 h, and the cell cycle analysis was performed using a FAC-Scan cytometry assay kit (BD Biosciences, San Jose, CA, USA) equipped with a single argon ion laser (488 nm). The DNA content of 1×10^4^ cells/analysis was monitored using the FACScalibur system. Cell cycle profiles were analyzed with ModFit software (Verity Software House, Topsham, ME, USA).

### *In vitro* wound-healing repair assay

To assess cell migration, cells were seeded into a 12-well culture dish and grown in DMEM containing 10% FBS to a nearly confluent cell monolayer. The cells were resuspended in DMEM medium containing 1% FBS, and the monolayers were carefully scratched using a 200 μL pipette tip. Cellular debris was removed by washing with PBS, and then the cells were incubated with a non-cytotoxic concentration of CoQ_0_ (0-5 μM) for 24 h. The migrated cells were photographed (100 × magnification) at 0 and 24 h to monitor the migration of cells into the wounded area, and the closure of the wounded area was calculated.

### Cell invasion assay

Invasion assays were performed using BD Matrigel invasion chambers (Bedford, MA, USA). For the invasion assay, 10 μL Matrigel (25 mg/50 mL) was applied to 8-μm polycarbonate membrane filters, 1 × 10^5^ cells were seeded to the matrigel-coated filters in 200 μL of serum-free medium containing CoQ_0_ (0-5 μM) in triplicate. The bottom chamber of the apparatus contained 750 μL of complete growth medium. Cells were allowed to migrate for 24 h at 37°C. After 24 h incubation, the non-migrated cells on the top surface of the membrane were removed with a cotton swab. The migrated cells on the bottom side of the membrane were fixed in cold 75% methanol for 15 min and washed 3 times with PBS. The cells were stained with Giemsa stain solution and then de-stained with PBS. Images were obtained using an optical microscope (200 × magnification), and invading cells were quantified by manual counting.

### Transient transfection of siRNA targeting β-catenin

Cells were transfected with β-catenin siRNA using Lipofectamine RNAiMAX (Invitrogen, Grand Island, NY) according to the manufacturer's instructions. For the transfection, cells were grown in DMEM containing 10% FBS and plated in 6-well plates to 60% confluence at the time of transfection. On the next day, the culture medium was replaced with 500 μL of Opti-MEM, and the cells were transfected using the RNAiMAX transfection reagent. For each transfection, 5 μL RNAiMAX was mixed with 250 μL of Opti-MEM and incubated for 5 min at room temperature. In a separate tube, siRNA (100 pM, for a final concentration of 100 nM in 1 mL of Opti-MEM) was added to 250 μL of Opti-MEM, and the siRNA solution was added to the diluted RNAiMAX reagent. The resulting siRNA/RNAiMAX mixture (500 μL) was incubated for an additional 25 min at room temperature to allow complex formation. Subsequently, the solution was added to the cells in the 6-well plates, for a final transfection volume of 1 mL. After incubation for 6 h, the transfection medium was replaced with 2 mL of standard growth medium, and the cells were cultured at 37°C. Then, the cells were co-incubated with or without CoQ_0_ (15 μM) for 24 h. The total protein levels in cells were determined by Western blotting.

### Animals

Female athymic nude mice (BALB/*c-nu*), 5–7 weeks of age, were purchased from The National Laboratory Animal Center (Taipei, Taiwan) and were maintained in caged housing in a specifically designed pathogen-free isolation facility with a 12 h/12 h light/dark cycle. The mice were provided rodent chow (Oriental Yeast Co, Tokyo, Japan) and water *ad libitum*. All of the experiments were conducted in accordance with the guidelines outlined by the China Medical University Animal Ethics Research Board. The animal protocols were approved by the Institutional Animal Care and Use Committee of China Medical University.

### Tumor cell inoculation

A total of 24 mice (5 to 7 weeks old) were randomly divided into four groups containing six animals per group. B16F10 cells (1 × 10^6^ cells) were mixed in a 200 μL matrix gel and then injected subcutaneously into the right hind flanks of nude mice. The experiments were performed using cells that had been passaged fewer than 20 times. After cell inoculation for 7 days, the treatment groups received CoQ_0_ (2 mg/kg b.w.) *via* intraperitoneal injection every 3 days for 15 days. The control group received the vehicle (PBS) only. To monitor drug toxicity, the body weight of each animal was measured every 3 days. Tumor volume in mice was compared with caliper measurements of tumor length, width and depth, and then calculated every 3 days using the formula: length × width^2^ × 1/2. On the 15^th^ day, all of the mice were sacrificed and the tumor tissues were removed and weighed. A veterinary pathologist examined the mouse organs, including the liver, lungs, and kidneys.

### Histopathological analyses

The biopsied tumor tissues were isolated to perform hematoxylin-eosin staining, immunohistochemical staining, and western blot. The tumor tissues were immediately fixed with 4% paraformaldehyde, sectioned, and stained using hematoxylin-eosin for light microscopy. For immunohistochemical staining, the non-specific binding was blocked with 1% (w/v) bovine serum albumin at room temperature for 1 h. The sections were then incubated with anti-β-catenin, anti-cyclin D1, anti-survivin, and anti-MMP-9 antibodies overnight at 4°C. The slides were incubated with biotinylated secondary antibody (Zymed Laboratories, South San Francisco, CA) for 20 min at room temperature. Finally, slides were incubated with avidin-biotin complex reagent and stained with 3,3′-diaminobenzidine according to the manufacturer's protocol (Histostain^®^-Plus Kit, Zymed Laboratories). For western blot, tumor tissues were homogenized in RIPA buffer containing 1% protease inhibitor cocktail and 1% phosphatase inhibitor cocktail (Sigma-Aldrich, St. Louis, MO) and samples (50 μg of protein) were subjected to electrophoresis on SDS gels (8-10%), transferred to a PVDF membrane. Then the remaining steps were followed as described earlier in this article.

### Statistical analyses

*In vitro* experiments are presented as mean and standard deviation (mean±SD). For *in vivo* experiments, mean data values are presented with standard error (mean±SE). All study data were analyzed using analysis of variance followed by Dunnett's test for pair-wise comparison.

## CONCLUSION

In conclusion, our data demonstrated that the efficacy of CoQ_0_ in cell growth inhibition, induction of apoptosis, and prevention of metastasis may be due to suppression of the Wnt/β-catenin signaling pathway in melanoma cells. Our results also highlight the importance of the Wnt/β-catenin and their transcriptional targets (including c-myc, survivin, cyclin D1, CDK4, and MMPs), which may serve as future targets for the development of therapeutic strategies against human melanoma. To the best of our knowledge, this is the first report that indicates the *in vitro* and *in vivo* anti-cancer potential of CoQ_0_ against malignant melanoma.
